# An Integrated Risk Management Model for Source Water Protection Areas

**DOI:** 10.3390/ijerph9103724

**Published:** 2012-10-17

**Authors:** Pei-Te Chiueh, Wei-Ting Shang, Shang-Lien Lo

**Affiliations:** Graduate Institute of Environmental Engineering, National Taiwan University, No.71 Chou-Shan Road, Taipei 106, Taiwan; Email: wei_ting@hotmail.com (W.-T.S.); sllo@ntu.edu.tw (S.-L.L.)

**Keywords:** risk assessment, source water protection area, quantitative risk, qualitative risk, land use

## Abstract

Watersheds are recognized as the most effective management unit for the protection of water resources. For surface water supplies that use water from upstream watersheds, evaluating threats to water quality and implementing a watershed management plan are crucial for the maintenance of drinking water safe for humans. The aim of this article is to establish a risk assessment model that provides basic information for identifying critical pollutants and areas at high risk for degraded water quality. In this study, a quantitative risk model that uses hazard quotients for each water quality parameter was combined with a qualitative risk model that uses the relative risk level of potential pollution events in order to characterize the current condition and potential risk of watersheds providing drinking water. In a case study of Taipei Source Water Area in northern Taiwan, total coliforms and total phosphorus were the top two pollutants of concern. Intensive tea-growing and recreational activities around the riparian zone may contribute the greatest pollution to the watershed. Our risk assessment tool may be enhanced by developing, recording, and updating information on pollution sources in the water supply watersheds. Moreover, management authorities could use the resultant information to create watershed risk management plans.

## 1. Introduction

Source water protection is the first barrier to drinking water contamination in the multi-barrier approach to safe drinking water [1]. Maintaining good water quality, based on watershed management of surface-water supplies, minimizes operating costs and reduces the degree of drinking water treatment required, the quantity of chemicals used during treatment, and the creation of treatment byproducts [2]. Due to public health considerations, several countries have established source-water assessment programs to analyze existing and potential threats to the quality of the public drinking water supply [2,3,4,5]. During the assessment process, the most important step is the determination of the susceptibility of the water supply to contamination [6]. Previous studies have developed approaches to identify those areas that are most critical for protection, using possible sources of contaminants that could affect water quality and the likelihood that they will cause a problem [4,6,7]. These approaches often include a risk assessment based on health-effects information.

Human health risks from any set of stressors can be evaluated based on the probability of an unwanted and harmful event occurring, and the severity of consequences should it occur. Furthermore, the probability and severity of risks are directly influenced by three major components: sources of potential risks, receptors of concern (human or other), and environmental pathways that potentially connect the receptors to the source inputs [1].

Currently, drinking water quality standards are used to regulate the treatment of water at plants in Taiwan. These standards are applied to water destined for human consumption and are established for select physical, chemical, microbiological, and radiological parameters [8]. For pathways specific to daily consumption, these standards are easily adopted in risk assessments to estimate the potential risks of each pollutant. However, conducting a health risk assessment for raw water is a complicated process due to the variety of direct and indirect pathways occurring for all activities supported in the watershed. Raw water in the watershed area may support multiple beneficial uses, such as aquatic life, recreation, and agriculture. Risk assessment is intended to be a transparent decision-making tool with clear assumptions, and the results of risk assessments are valuable for informed risk management decisions. Therefore, establishing a risk assessment model for source water could enhance the protection of water quality in the watershed and help to prioritize management actions.

Quantitative and qualitative risk assessments have been applied to some watershed management plans in order to acquire priority information for implementing protection actions [9]. Quantitative risk is often used for pollutants with complete information on hazard threshold values, such as carcinogenic chemicals. On the other hand, qualitative risk assessment is often used for pollutants without specific threshold values for negative effects on human health, such as nutrients in the watershed. The application of such qualitative models for nutrient management is often used to identify areas of greatest potential nutrient export or critical source areas. For watershed management, quantitative risk could be used to illustrate past and current conditions of water quality by estimated exposure, and qualitative risk could be used to assess the potential risk of all possible events, including existing facilities and activities, together with future, estimated loadings. However, there has been little discussion about applying both quantitative and qualitative risk assessment methods to examine current water quality and investigate potential pollutant sources. To fill the gap in watershed management, an integrated model of quantitative risk indicators and qualitative event analyses were implemented to provide complete information for watershed management and protect source water. This paper presents the development of models to evaluate the risk to humans of drinking water sourced from surface water supplies. The results are intended to guide informed risk management decisions for utility managers who have water quality targets to achieve. A case study of the Taipei Source Water Area (TSWA) in Northern Taiwan is also presented.

## 2. Materials and Methods

### 2.1. Study Area

**Figure 1:**
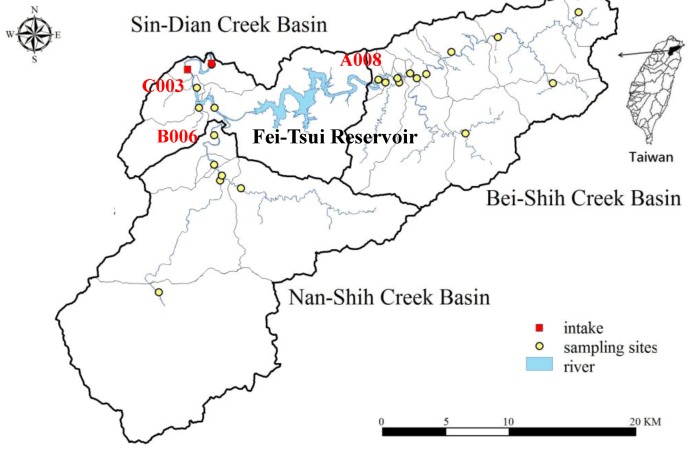
Study area.

The TSWA is a unique area that has been delineated for protecting source water in Taiwan. It covers an area of 697.57 km^2^ and encompasses three basins: Bei-Shih, Nan-Shih, and Sin-Dian Creek Basins, as shown in Figure 1. The Fei-Tsui reservoir, which provides drinking water for around five million people in northern Taiwan, is located in the Bei-Shih Creek Basin. Land-use activities such as mining, forestry, and recreation are limited by legislation in this area. Its water quality is classified as Class A, according to the Taiwan EPA surface water quality standards for public water use.

This study divided the TSWA into 22 sub-watersheds (Figure 1) based on the sampling sites and their drainage areas, which were established using the digital elevation model tool in ArcInfo 9.3. Sampling sites which had been established in previous monitoring programs included areas of high population density, plus up- and downstream tributaries connected to the main body of water. Water sampling was conducted monthly; samples were manually collected and analyzed by qualified laboratories using standard methods. For identifying the health significance of potential pollutants and the pathways along which these pollutants move to watersheds, both historical and new results from the various sites were used in the development of qualitative and quantitative components in this study.

Land-use data were obtained from the National Land Surveying and Mapping Center, Taiwan [10], and imported into the sub-watershed map of the study area. The Center conducted a nationwide land-use investigation during 2006–2008 using aerial photography and SPOT-5 satellite imagery. Table 1 lists the areas and percentage areas of different land-use types in the TSWA. More than 90% of the total area was forested. The percentage of forest in both the Bei-Shih and Nan-Shih Creek Basins, more than 87%, was higher than in Sin-Dian Creek Basin; in contrast, the built-up area in Sin-Dian Creek Basin of 9.1% was higher than in either Bei-Shih or Nan-Shih Creek Basin. The Sin-Dian Creek Basin also occupied larger farmland of 12.8% than in the other two basins. Of the three, Bei-Shih Creek Basin had the largest area of non-irrigated farmland without vegetative cover.

**Table 1:** Area and percentage area of different land-use activities in the TSWA.

Land-use activities	Bei-Shih Creek		Nan-Shih Creek		Sin-Dian Creek		Total	
	Basin		Basin		Basin			
	Area	Area	Area	Area	Area	Area	Area	Area
	(km^2^)	(%)	(km^2^)	(%)	(km^2^)	(%)	(km^2^)	(%)
Built-up area	4.38	1.4	1.75	0.5	4.13	9.1	10.26	1.5
Forest	277.14	87.5	328.65	98.0	33.66	74.6	639.45	91.7
Farmland	18.84	5.9	1.44	0.4	5.77	12.8	26.05	3.7
Non-irrigated farmland without vegetative cover	4.24	1.3	1.02	0.3	0.12	0.3	5.38	0.8
Water area	12.39	3.9	2.58	0.8	1.46	3.2	16.43	2.3
Total	316.9	100	335.44	100.0	45.14	100	697.57	100.0

### 2.2. The Quantitative Risk Assessment Model

The quantification of risk for the Taipei Metropolitan drinking water supply was carried out by comparing the measured levels of a potential pollutant that humans might be exposed to against the threshold level for negative consequences of that pollutant. The indicator, the hazard quotient (HQ), is the ratio of the expected exposure to the estimated safe threshold (Equation (1)). Results from past sampling programs in the TSWA were used to estimate human exposure. A hazard quotient greater than 1.0 implies that the degree to which humans are exposed exceeds allowable limits established to prevent undesired effects:


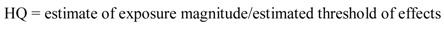
(1)

In this study, past sampling data of the TSWA were used for estimating human exposure, and the established water quality criteria for Class A surface water (Table 2) were used as safe thresholds. Although source water might not be consumed directly by humans, conservative values are generally considered for risk assessment. Drinking Water and Surface Water Classifications and Water Quality Standards [8] were compared to determine the thresholds for calculating the HQ in this study (Table 2). In this study, the standards for Class A were adopted to represent the potential for pollution in the watershed because the water quality parameters were consistent with available monitoring data for watershed management practices.

**Table 2:** Water quality standards for Class A surface water *vs*. drinking water.

Parameters	Standard Values	
	Surface Water Bodies	Drinking Water
	Class A *	Quality Standards
pH	6.5–8.5	6.0–8.5
DO (mg/L)	≥6.5	**
BOD (mg/L)	≤1	--
Solids (mg/L)	≤25 (suspended solids)	≤ 500 (total dissolved solids)
*E. coli*	≤50	≤6
(CFU/100 mL)		
NH_3_-N (mg/L)	≤0.1	≤0.1
TP (mg/L)	≤0.02	--

* Class A water bodies may be used for Class 1 public water, swimming and as other class water bodies. Class 1 public water means water sources that have been disinfected and can be used as public water supplies. ** Not specified.

### 2.3. The Qualitative Risk Assessment Model

A qualitative risk assessment model was developed to identify sub-watersheds within the study area that were at greatest risk of impaired water quality. The model required information on watershed attributes and land use.

The qualitative risks were analyzed using a matrix that considered the consequences of a hazardous event and the likelihood of identified hazards causing harm in exposed populations over a specified period. Three components were considered to frame the assessment matrix (Table 3):

1. Source characteristics (S) were used for hazard identification. Higher priority was given to possible sources of human pathogens (e.g., parasites, bacterial, and viral diseases); lesser priority was given to sources of turbidity, chemical contamination, or nutrients.
2. Proximity to water (P) was used to evaluate the pathways introducing pollutants to source water.
3. The spatial extent (E) of an activity, land use or other issue was used to assess both source and transport pathways in the watershed.

These three factors were ranked into four levels for each sub-watershed, as shown in Table 3. The classification of risk levels for the three factors was established in the research of watershed management plan for City of Whitehorse, Canada [1]. The product of the levels of the three components (R = S × P × E) was classified into four risk levels, low (≤6), medium (≤9), high (≤16), and very high (>16). The levels were based on comparative risks, and provide an approximation of the relative, not absolute, differences between risk levels in order to sort activities and issues by risk potential. Therefore, a maximum value of 64 does not imply a 4-fold greater risk potential than an activity with a value of 16 [1]. Since a detailed inventory of pollution sources was not available, this qualitative model tends to screen potential issues in for further investigation, rather than screening them out.

**Table 3:** The qualitative risk assessment model for prioritizing risk factors for the TSWA source water protection.

Factors	Source characteristics (S)	Proximity to water (P)	Spatial extent (E)	Risk level
Description	The characteristics of pollution source	The distance from the closet source water to area or activity of interest	The percentage of effective sub-watershed involved with the area or activity of interest	The higher value means higher priority of risk potential
Classification	Major source of possible contamination by pathogens	0-50 m	1-10%	4
	Minor source of possible contamination by pathogens	50-250 m	0.1-1%	3
	Major source of possible non-pathogenic contaminants	250-1,000 m	0.01-0.1%	2
	Minor source of possible non-pathogenic contaminants	>1,000 m	<0.01%	1

Watershed attributes that can influence water quality include human land uses as well as natural characteristics. Several attributes were included in the qualitative risk assessment in this study (Table 4):

▪ Sub-watershed slope
  Average sub-watershed slope was considered because it provides a generalized indication of slope stability and potential for runoff; steep terrain increases the chance of landslide or debris flow and has the potential to deliver more surface runoff to streams. Average sub-watershed slope was determined from digital elevation model mapping data and calculated in this study using ArcGIS 9.3.
▪ Agriculture
  In the Bei-Shih Creek basin, tea-growing was the major source of potential contamination resulting from its use of fertilizers and pesticides. Although organic farming was initiated over a decade ago, most growers in the area continue to use traditional methods.
▪ Recreation
  Although the TSWA is a delineated protection area, recreational activities such as camping and playing in the water are popular during the summer. In addition, the Nan-Shih Creek Basin is a famous and scenic area with hot springs, and there are dozens of inns and restaurants around the creek. According to the investigation of Lin *et al.* [11] untreated bathing water discharged into Nan-Shih Creek may violate river water quality standards for ammonia, nitrogen, and total coliforms during peak tourist seasons. Recreation is therefore considered as a potential risk to the water environment of Nan-Shih Creek Basin. As the scale of recreation activities in the study area is often small, the risk level of spatial extents of recreation was assumed to be 1. On the other hand, almost all recreation activities were near to the streams, and the risk level of proximity to water was assumed to be 4.
▪ Built-up areas and urban development
  Urban development can have significant impacts on water quality. Runoff in urban areas generated from roofs, parking lots, and streets can contribute fertilizers, grease, organic contaminants, heavy metals, pesticides, salt, sediment, nutrients, pathogens, and fecal matter to receiving bodies of water [7]. Urban areas generate effluents from sewage treatment plants and industrial processes, which may be discharged directly into nearby rivers. The extent of urban development within each sub-watershed was calculated by dividing the built-up area by the total land area.

**Table 4:** Sub-watershed attributes.

Basin	Sub-watershed	Average slope (degree)	Total	Tea-growing		Built-up	Population	
			Area	area		area	density	
			(km^2^)	(km^2^)	%	Area(km^2^)	%	(people/km^2^)
Bei-Shih Creek Basin	A001	21.66	42.89	0.789	1.84	0.53	1.24	19
	A002	22.41	12.01	1.707	14.21	0.64	5.33	123
	A003	25.45	36.87	0.109	0.30	0.2	0.54	8
	A004	25.85	43.21	1.407	3.26	0.43	1.00	43
	A005	23.31	1.13	0.145	12.82	0.03	2.65	301
	A006	23.58	24.96	1.518	6.08	0.24	0.96	33
	A007	25.32	2.15	0.162	7.55	0.06	2.79	34
	A008	26.71	1.02	0.010	1.01	0	0.00	11
	A009	15.46	11.97	0.170	1.42	0.08	0.67	13
	A010	23.34	21.12	0.064	0.30	0.04	0.19	12
	A011	25.35	26.37	2.573	9.76	0.6	2.28	41
	A012	21.99	3.04	0.238	7.83	0.14	4.61	25
Nan-Shih Creek Basin	B001	30.13	163.99	0.014	0.01	0.23	0.14	3
	B002	30.22	66.36	0.184	0.28	0.92	1.39	31
	B003	28.62	83.78	0.005	0.01	0.19	0.23	14
	B004	24.65	0.42	0.001	0.29	0.03	7.14	117
	B005	26.56	2.69	0.010	0.38	0.08	2.97	99
	B006	28.13	18.21	0.115	0.63	0.3	1.65	110
Sin-Dain Creek Basin	C001	26.14	19.66	0.174	0.89	0.27	1.37	59
	C002	24.26	90.27	1.621	1.80	1.39	1.54	55
	C003	22.06	13.68	0.411	3.00	2.44	17.84	249
	C004	19.72	11.81	0.830	7.03	1.42	12.02	618

## 3. Results and Discussion

### 3.1. Quantitative Risk

Using the means and maxima of past sampling data from 2008 to 2010 to estimate exposure, and the Class A water quality standards as the thresholds, we calculated the HQs of those three sites located nearest to the inlets of treatment plants (Table 5). The results indicated that the risk of total coliforms was the greatest of all parameters since the HQs for all three sites were far greater than 1.0. The value for Bei-Shih Creek was higher than for the others. Coliform groups by themselves are usually not pathogenic; they are indicator organisms, which means they may indicate the presence of other pathogenic bacteria. Pathogens are typically present in such small amounts it is impractical monitor them directly. Although the drinking water standards for total coliforms is less than 6 MPN/100 mL (Table 2), which can generally can be achieved after a chlorinated disinfection process, the total coliforms in the watershed often exceeded the Class A standard, revealing that potential microbiological pollution sources exist in the watershed. This may have minor consequences for humans drinking the raw water directly, but only a low risk resulting from contact during other activities. Total coliforms may come from animal agriculture, wildlife, recreational activities, and urban development, or may fluctuate naturally. However, detailed investigations such as microbiological source tracking are necessary to prevent other pollutants such as manure and fertilizer that might be discharged into the watershed in combination with these fecal contaminants.

**Table 5:** Screen level evaluation of human health risks from TSWA based on source water monitoring data.

Stations *	Substance	No. data points (No. of detected results)	Average (Range of Values)	Max. Value	Class A water quality standard	Hazard Quotient	
						(Avg.)	(Max.)
A008	Total coliforms	32 (29)	2,741.0 (30-58,000)	58,000	50	54.82	1,160.00
	Total phosphorus	32 (21)	0.028 (0.013-0.093)	0.093	0.02	1.42	4.65
	Ammonia nitrogen	32 (20)	0.048 (0.01-0.2)	0.2	0.1	0.48	2.00
	Suspended solids	32 (29)	5.45 (1.3-36.1)	36.1	25	0.22	1.44
	Biochemical oxygen demand	32 (32)	1.06 (0.3-3.8)	3.8	1	1.06	3.80
	Dissolved oxygen	32 (32)	7.49 (6.5-8.8)	8.8	6.5	-	-
	pH	32 (32)	7.51 (6.7-8.5)	8.5	6.5-8.5	-	-
B006	Total coliforms	32 (32)	3,048.0 (95-37,000)	37,000	50	60.96	740.00
	Total phosphorus	32 (29)	0.049 (0.015-0.287)	0.287	0.02	2.45	14.35
	Ammonia nitrogen	32 (22)	0.035 (0.01-0.17)	0.17	0.1	0.35	1.70
	Suspended solids	32 (29)	30.02 (1.1-345)	345	25	1.20	13.80
	Biochemical oxygen demand	32 (32)	0.94 (0.4-3.3)	3.3	1	0.94	3.30
	Dissolved oxygen	32 (32)	7.78 (6.6-8.6)	8.6	6.5	-	-
	pH	32 (32)	8.11 (7-9)	9	6.5-8.5	-	-
C003	Total coliforms	36 (36)	1,111.1 (25-7,500)	7,500	50	22.22	150.00
	Total phosphorus	35 (29)	0.093 (0.015-0.58)	0.58	0.02	4.65	29.00
	Ammonia nitrogen	36 (26)	0.135 (0.01-1.7)	1.7	0.1	1.35	17.00
	Suspended solids	36 (35)	15.66 (0.6-126)	126	25	0.63	5.04
	Biochemical oxygen demand	36 (36)	1.04 (0.3-6.9)	6.9	1	1.04	6.90
	Dissolved oxygen	36 (36)	7.85 (6.3-10.2)	10.2	6.5	-	-
	pH	36 (36)	7.54 (6.5-8.7)	8.7	6.5-8.5	-	-

* See Figure 1 for the location of stations.

The HQs for total phosphorus (TP) were also greater than 1.0 in analyses using both average and maximum values as the estimated exposures. TP is considered to be associated with toxins released by cyanophytes. Comparing the values for the three sites, the TP HQ of Sin-Dian Creek was higher than that of the other two basins. The limit of TP (≤0.020 mg/L) for Class A surface water standards corresponds to rivers in near pristine condition, and the value is difficult to achieve even for undeveloped, natural forestry. Many risk indicators have been developed to assess the risk of phosphorus loss from agricultural land to surface water. Phosphorus-related impacts on water bodies are a major environmental and economic problem, and are manifested as an increase in primary production, which leads to eutrophication and even toxic blue-green algal blooms. However, there is little information regarding the correlation between phosphorus concentrations in raw water and water treatment loading. The high HQs for phosphorus in the study area need further research in the form of a review of surface water standards (thresholds for designated uses) based on specific source properties and characteristics of the receiving systems. Ranking potential phosphorus sources by applying qualitative risk analysis, and reducing these sources before they are introduced into the watershed, could supplement the shortcomings of quantitative analysis.

The HQs for ammonia nitrogen were greater than 1.0 only when using the maximum as the threshold. Ammonia accumulates easily in aquatic systems because it is a natural byproduct of fish metabolism. Human activities, such as playing in the water and camping, may also increase the nitrogen loading. Agriculture activities, especially from the spreading of fertilizer and manure are also considered as major sources of ammonia nitrogen. However, according to sampling data at least, hot spring effluents did not increase the nitrogen concentration in the Nan-Shih Creek. 

**Figure 2:**
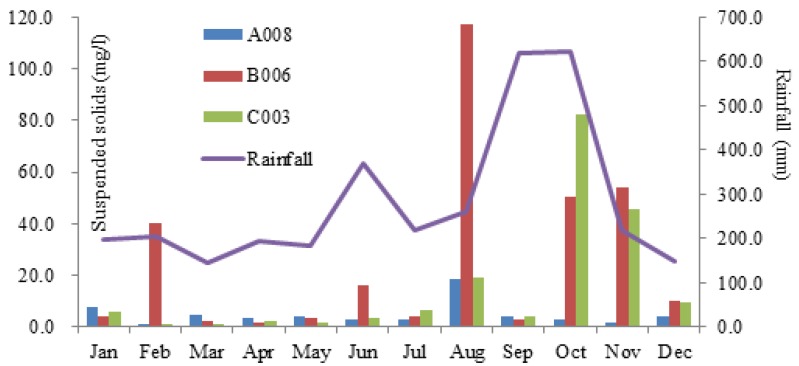
The trends of suspended solids concentration and rainfall in the Taipei Source Water Area.

Suspended solids (SS) is a parameter that may be influenced by rainfall and urban development. The existence of SS in source water may reduce the efficiency of chlorination in treatment process. Maximum HQs for all three sites were greater than 1.0, while that in Nan-Shih Creek was higher the others. Although the correlation between average monthly SS concentration and monthly rainfall was not significant, due to fast flowing streams and hydropower operations in the basin, Figure 2 still shows similar trends of summer increases for SS concentration and monthly rainfall.

The average biochemical oxygen demand (BOD) HQs of Bei-Shih and Sin-Dian Creeks were greater than one, and indicated potential anthropogenic pollution in these two basins. Other parameters, such as dissolved oxygen and pH, were always within a safe range and indicated a low risk to human health. Nevertheless, the variation of dissolved oxygen and pH may cause chemical reactions that result in changes to other parameters, such as the dominant forms of nitrogen in the water, and these fluctuations should still be monitored.

The characteristics of each parameter for the other 19 sites were similar to those of the three sites nearest to the intakes discussed above, and showed no significant trends along the river from upstream to downstream.

### 3.2. Qualitative Risk

A list of activities and situations for the three basins was developed based on concerns about the watershed and drinking water supply (Table 6), using watershed attributes mentioned in Section 2.3. Previous research on the study area found tea-growing to be the greatest source of risk in the Bei-Shih Creek Basin and recreational activities (including bathing and lodging in the hot spring scenic area, and playing in the water) to be the major sources of pollution for the Nan-Shih Creek Basin [12]. For the Sin-Dian Creek Basin, residential development and recreational activities along the stream caused concern about potential pollution. In addition, on-site investigations and the results of quantitative risk analysis indicated that the risk of landslides and high levels of suspended solids should also be considered for Nan-Shih Creek Basin.

**Table 6:** Summary of source water risk characterization for the TSWA.

Source Type	S: Source characteristics-Risk index (1-4) *	P: Proximity to Water-Risk index (1-4) *	E: Spatial extent-Risk index (1-4) *	Max. Overall risk index Product = S × P × E **
**Possible sources of pathogens, chemicals, and nutrient inputs to Bei-Shih Creek Basin**				
Tea plantation near the Bei-Shih Creek	2	1-4	1-4	18
Recreational activities around the Creek and reservoir	1	1-4	1-4	8
**Possible sources of pathogens, chemicals, and nutrient inputs to Nan-Shih Creek Basin**				
Recreational activities and wastewater from the hot spring	2	1-4	1-4	8
Recreational activities around the Creek and reservoir	1	1-4	1-4	8
Slides from mountain near the riparian zones	1	1-4	1-4	8
**Possible sources of pathogens, chemicals, and nutrient inputs to Sin-Dan Creek Basin**				
Residential, other developments around the inlet of treatment plant	3	1-4	1-4	8
Recreational activities around the Creek and reservoir	1	1-4	1-4	8

* See Table 3 for a description of risk index rankings. ** Maximum risk score for sub-watersheds, see Figure 5.

**Figure 3:**
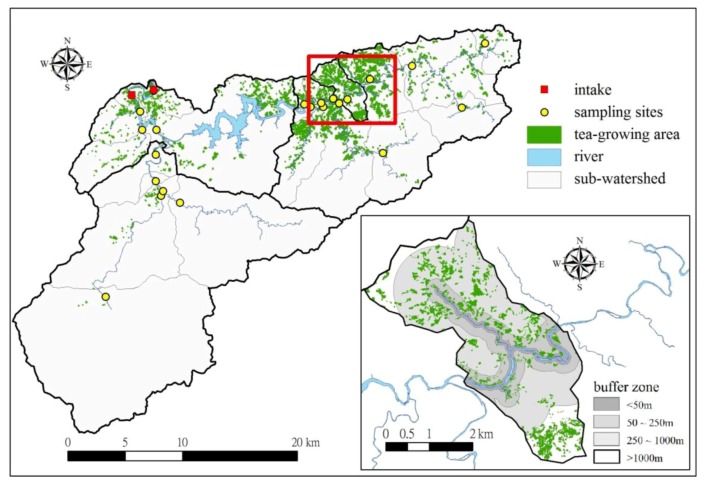
Tea-growing distribution in the TSWA and sub-watershed A002.

Results from this study indicated that intensive tea-growing was the greatest risk for the TSWA due to the location of plantations in or near the riparian zone and their occupation of a relatively wide area in some sub-watersheds. The distributions of the tea-growing areas are shown in Figure 3. The sub-watershed A002 had the highest ratio (14.21%) of land used for tea-growing and Table 7 illustrates the spatial extent and proximity for each risk level. According to the study of Hirono *et al.* [13], tea cultivation often involves the application of large quantities of nitrogen as fertilizer, resulting in high nitrate-nitrogen concentrations in the groundwater around these areas. The results of that also indicated that water systems surrounding tea-growing areas are likely to be acidified; therefore, the risks from tea-growing in the source water area should be emphasized. Of the source characteristics for tea-growing, nutrient pollution was considered a major category and risk level 2 was selected. The maximum overall qualitative risk derived from the three factors (Table 6) was 18 and tea-growing was classified at the level of “very high” risk.

**Table 7:** The risk level of sub-watershed A002.

Sub-watershed	Area (m^2^)	Tea-growing area		Tea-growing area in buffer zones (S = 2)			
		Area (m^2^)	%	0-50 m	50-250m	250-1,000m	>1,000m
				(P = 4)	(P = 3)	(P = 2)	(P = 1)
A002	12,012,334	1,707,087	14.2%	71,267	374,034	810,881	450,905
				0.6% (E = 3)	3.1% (E = 4)	6.8% (E = 4)	3.8% (E = 4)
R = S × P × E				24	24	16	8
Ave. R				18			

Note: S = Source, P = proximity, E = Extent, R = risk.

**Figure 4:**
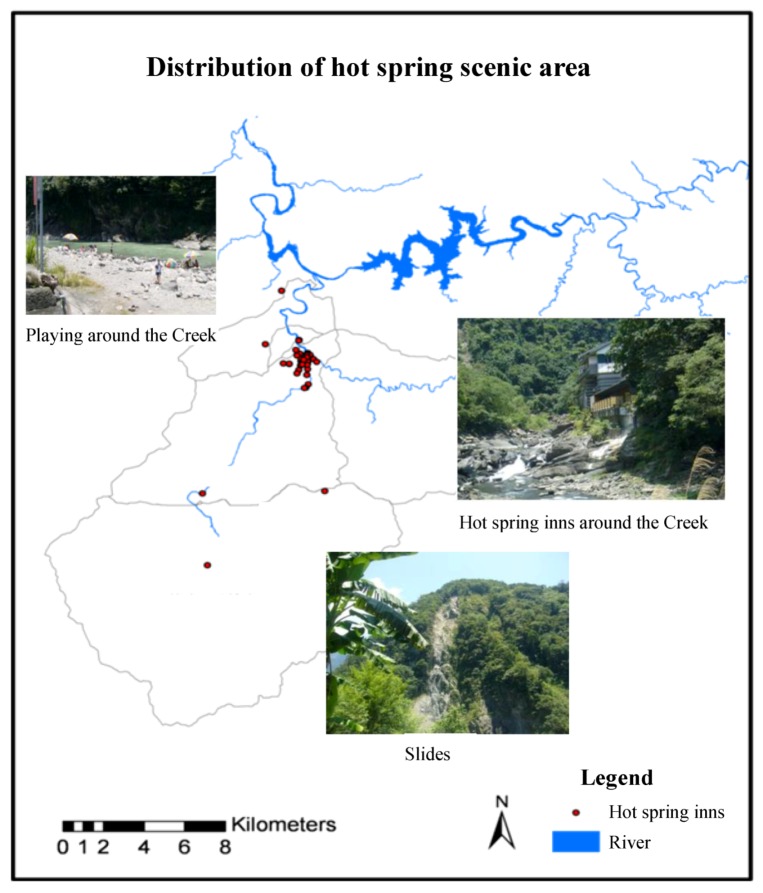
The potential pollution source of Nan-Shih Creek Basin.

Recreational activities around the creek and reservoir were a common source of potential pollution for the three basins. All recreational activities were classified as minor sources of possible non-pathogenic contaminants, and all were located close to the bodies of water but often sparsely distributed. Therefore, a risk level of “low” or “medium” was derived from the qualitative risk model (Table 6). However, hot spring wastewater from bathing and playing was classified as a “medium” risk source due to the more complex nature of this wastewater. Figure 4 presents the location of hot spring scenic area in the Nan-Shih Creek Basin.

In the Nan-Shih Creek Basin, landslides from mountains near the riparian zones were included as a potential risk event. The slopes range between 24–30 degrees for the six sub-watersheds and were considered steep enough to generate slides (Table 4). Confirming this possibility, monitored levels of suspended solids showed an increasing trend over the rainy season.

**Figure 5:**
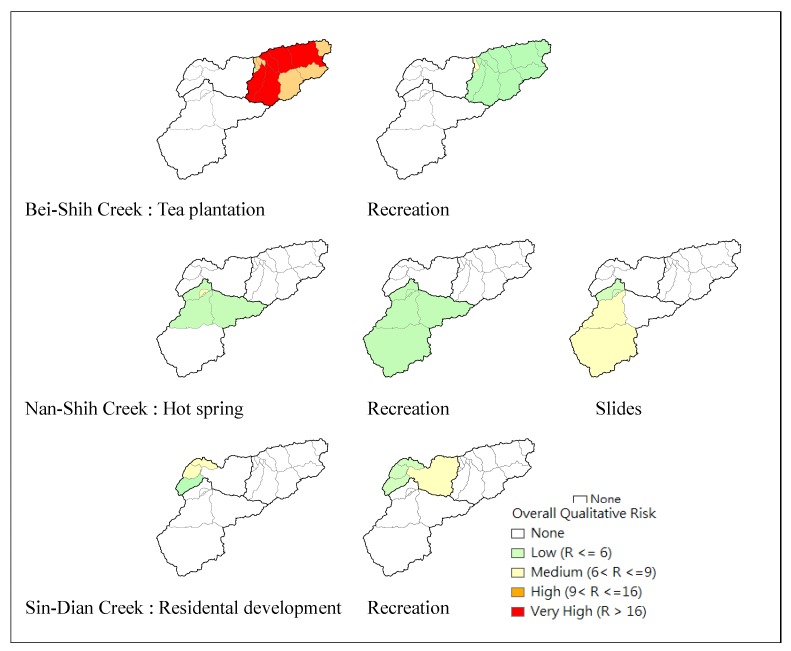
Qualitative risk assessments.

In the Sin-Dian Creek Basin, residential development was considered a pollution source. The percentage of built-up area was relatively high in the TSWA and the activities of residents were concentrated along the riparian zone. All potential contamination sources considered in the qualitative risk analysis were ranked according to the risk level of factors and the results are illustrated in Figure 5.

Summarizing the potential risk of each potential pollution source and taking an average of the risk levels, there are five sub-watersheds of the Bei-Shih Creek Basin classified as very high risk resulting from intensive tea plantation.

### 3.3. Overall Risk Analysis and Management

Integration of the analyses of quantitative and qualitative risks revealed total coliforms and total phosphorus to be the top two parameters of concern for source water. Intensive tea-growing in the Bei-Shih Creek Basin, together with all types of recreation around the creeks and reservoirs in all three basins, are highly ranked risk sources for humans that drink water supplied by this area. Suspended solids increased in the summer season in the Nan-Shih and Sin-Dian Creek Basins primarily due to the natural factors of sub-watershed slopes and rainfall events. 

Watershed management plans, based on land use restrictions, seek to reduce or eliminate sources of potential risk before they are introduced into the watershed. In addition, riparian zone protection is intended to decrease the potential exposure pathways within a watershed, so that the magnitude of movement from source to drinking water intake point is effectively reduced. Isolating all possible pollution sources is the most direct control measure for reducing the risk to drinking water while the watershed remains in pristine condition and has no residential development. However, the TSWA is located around the Taipei metropolitan area, with a large population: substantially changing existing land use and activity patterns is not practical. Therefore, best management practices, riparian zone restoration, and restrictions to the spatial extent of activities are practicable strategies that can be implemented quickly. Maintaining cleanliness in scenic areas and public education about recreational activities in source water protection zones would also constitute effective control measures.

The present integrated risk management model can be applied to the susceptibility analysis for a public water system which consists of determining the target pollutants and level of threat within the watersheds. Furthermore, a watershed management plan can be implemented based on the indicator of hazard quotients and the determined risk levels for sub-watersheds.

## 4. Conclusions

Various watershed attributes and types of land-use activities have the potential to degrade the water quality within watersheds. Quantitative and qualitative risk assessments, which consider watershed attributes that influence water quality, as well as the threshold of sensitivity of humans that drink the water, could be valuable tools for identifying areas at high risk of degraded water quality. The results could be used as part of a screening tool to prioritize watersheds for assessment, particularly in systems that lack detailed inventories of potential pollution contributions. If target activities are identified through a watershed assessment, further monitoring and investigation could be undertaken to identify the quantitative risks of pollution.

Future risk analyses should continuously revise the process maps of water quality hazards using updated knowledge of contaminants. In addition, detailed assessment of “high” risk hazard events that influence water quality is essential for preemptive reduction of source water risk. The value of this risk assessment tool relies on continuously developing, recording, and updating information on pollution sources in the water supply watersheds, and management authorities should establish risk management plans based on such information.
